# An Ethnobotanical Study of Wild Edible Plants in Tach Gayint District, South Gondar Zone, Amhara Region, Northwestern Ethiopia

**DOI:** 10.1155/2023/7837615

**Published:** 2023-06-27

**Authors:** Yalew Yiblet, Endale Adamu

**Affiliations:** ^1^Department of Biology, Mekdela Amba University, P.O. Box 32, Tulu Awlia, Ethiopia; ^2^Department of Biology, College of Natural and Computational Sciences, Debre Tabor University, P.O. Box 272, Debre Tabor, Ethiopia

## Abstract

The purpose of this study was to carry out an ethnobotanical and ethnopharmacological investigation on wild edible plants and their value to households in the Tach Gayint district of South Gondar Zone of northwestern Ethiopia. A total of 175 informants (56 women and 119 males) were interviewed for ethnobotanical data, and 25 of them were key informants. Data collection techniques included semistructured interviews, guided field walks, and focus group discussions. Quantitative analytical tools were employed for ethnobotanical methods including preference ranking and direct matrix ranking techniques to analyse the data. 36 species of wild edible plants have been identified in the study area. Of these plant species, shrubs account for 15 (42%), followed by herbs 13 (36%) and trees 8 (22%). Regarding the edible parts, fruits account for 19 (53%) followed by young shoots, leaves, and flowers, 4 (11%) for each. These plant species are eaten raw (86%) or cooked (14%) and most are collected by younger people for herding cattle. According to the preference ranking analysis, the fruit of *Opuntia ficus-indica* is the most preferred plant species because its sweet taste. Although *Cordia africana*, the most commonly used multipurpose wild edible plant species, is mostly exploited due to human activity, activities such as the production of charcoal, the gathering of firewood, the construction of homes, and the use of agricultural tools all played a significant role in the plant's eventual extinction. In the study area, agricultural expansion was the main cause of putting wild edible plants in danger. It is best to cultivate and manage edible plants in a backyard garden and to perform more research on popular edible plant species.

## 1. Introduction

Rural communities in developing countries rely on edible wild plants to meet their nutritional demands during times of food scarcity. According to studies by [1], wild edible plants (WEPs) are predominantly consumed as addition to conventional diets in numerous African locations. Food plants are occasionally eaten for their health benefits, and several species are frequently used as herbal remedies in traditional phytotherapy to treat a variety of illnesses and problems [2]. The World Health Organization (WHO) reports that conventional herbal medicines are used by 80% of people in developing countries [3]. Since ancient times, indigenous people around the world have used a variety of herbs to treat burns and other ailments. They contain terpenoids, tannins, alkaloids, flavonoids, essential oils, phenolic compounds, saponins, and fatty acids, which have a wide range of pharmacological potential, including anticancer, antidiabetic, and antimicrobial effects, as well as cosmetic properties [4].

Plants are a significant source of active antioxidants that protect the body from many oxidative stress, according to [5] reports. Pharmacotherapy, especially for the treatment of cancer and infectious disorders, has been dominated and significantly influenced by natural chemicals and their biosynthetic modifications [6]. In Ethiopia, there are about 6000 species of higher plants, 10% of which are endemic. The country is considered a hotspot for biodiversity, origin, and diversification of a significant amount of domesticated food plants, as well as those of their wild relatives. According to ethnobotanical studies conducted in Ethiopia, the plant species, *Echinops kebericho*, *Kalanchoe petitiana*, *Lippia adoensis*, and *Aloe adigratan* [7], *Clematis longicauda*, *Millettia ferruginea*, and *Pycnostachys abyssinica* [8], and *Aloe yavellana*, *Erythrina brucei*, *Solanecio gigas*, and *Thunbergia ruspolii* [9], were reported as endemic medicinal plants and *Dioscorea praehensilis* [10] was reported as a wild edible plant in the flora.

Many rural populations are very skilled in making use of plant resources, according to a certain research conducted in Ethiopia [11, 12]. In this way, older members of the community are frequently the most significant sources of plant knowledge [13]. Native people prefer WEPs not only for their regular nutritional content but also for their ability to replace a variety of food gaps, as well as for their numerous applications to the health of humans, animals, and the environment [14]. As reported by Lulekal et al. [15], 413 WEPs are consumed in Ethiopia from food insecurity areas. For instance, in southern Ethiopia's Konso, Derashe, and Burji special districts, WEPs appear to be better because of the periodic climate shocks that impede agricultural productivity and cause food shortages [16]. Similarly, the consumption of WEP is common in northern Ethiopia, such as *Adansonia digitata*, *Balanites aegyptiaca*, *Carissa spinarum*, *Cordia africana*, *Tamarindus indica*, *Ximenia americana,* and *Ziziphus spina-christi* [17]. Most of the WEPs are consumed by children specially, the fruits of *Ficus* spp., *Carissa spinarum*, and *Rosa abyssinica*, among others. In Ethiopia, the consumption of wild foods and their importance in meeting nutritional needs in rural communities, as well as socioeconomic, cultural, and traditional aspects, is still underreported and receives little attention [18]. The Tach Gayint district is one of the food insecure areas of the region. Therefore, extensive ethnobotanical research is necessary to gather information on plants and related indigenous knowledge for conservation and sustainable use. There are relatively few or no studies on traditional knowledge and practice with WEP species in remote areas of Ethiopia, where its use is quite common in times of surplus and hunger. Therefore, the objective of this study was to assess and gather indigenous knowledge used by the people of the Tach Gayint district, as well as to list the WEP species that were consumed by the locals.

## 2. Materials and Methods

### 2.1. Description of the Study Area

**Figure 1:**
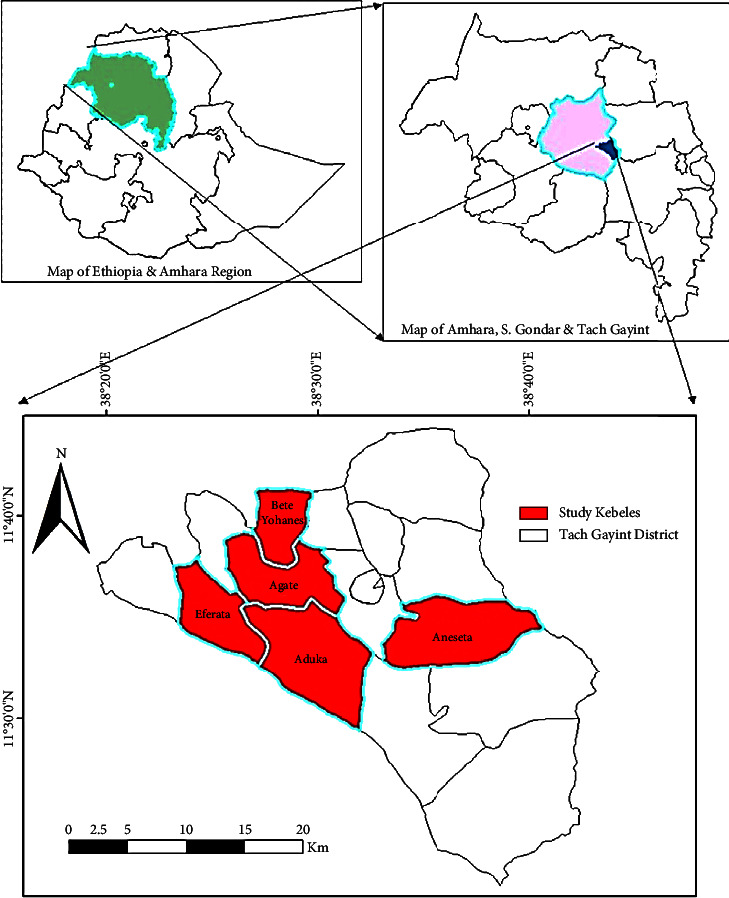
Map of the study area: a map of Tach Gayint with the study kebeles highlighted in red.

The study was conducted at the Tach Gayint district of South Gondar Zone of the Amhara Regional State of northwestern Ethiopia located within 1129′59.99″–1115′36″ and 3814′60″–3837′42″ latitude and longitude, respectively (Figure 1). The Tach Gayint district is located at a distance of 766 km from Addis Ababa (capital city). It is 197 km from Bahir Dar city (regional city) and 100 km from Debre Tabor (zone town). Elevation varies between 750 and 2800 metres above the sea level. The district is bounded on the north by the Lay Gayint, on the east by North Wollo, on the West by Simada, and on the South by Lay Gayint [19]. The district has a total of 16 kebeles administrations and a total population of 105,441 people, with 59,823 men and 45,618 women. 92.2% of the Tach Gayint residents are Orthodox Christians, 7.7% are Muslims, 0.001% are Catholics, and 0.001 percent are the members of a protestant church. Amhara makes up 99.9% of the population, indicating that the residents of Tach Gayint are practically all from the same ethnic group. Dega, Woina Dega, and Kola are the three agroecological zones in the research area. Dega (also known as Wurch in the local tongue) is a highland agroecological zone with a cool climate. Woina Dega is a subtropical agroecological zone with a moderate climate and is known locally as humid. Kola, which means “hot” in the native language, is an agroecological zone with hot climatic conditions [20].

The research area is one of the most drought-prone and susceptible parts of the region, and land resources can be used for a variety of socioeconomic and subsistence needs. Approximately 74% of the land used for cereals, annual and perennial crops, grazing, forests and shrubs, settlements, and wastelands make up the remaining land use. There is just one rainy season (referred to locally as “kiremt”), which is essential for the growth of both long-cycle and short-cycle crops. Crop production is completely dependent on rainfall. A mixed farming system, which produces crops through multiple cropping on a small amount of land intensively, has been utilised in the region. Teff (*Eragrostis tef*), wheat (*Triticum aestivum*), barley (*Hordeum vulgare*), beans (*Vicia faba*), wasera wheat (*Triticum aestivum*), and barley (*Hordeum vulgare*) grown together are the main food crops grown in the area. Continuous cropping has traditionally been accomplished by crop rotation, which alternates the production of cereal crops with the cultivation of legume crops to sustain soil fertility [21].

### 2.2. Climate

**Figure 2:**
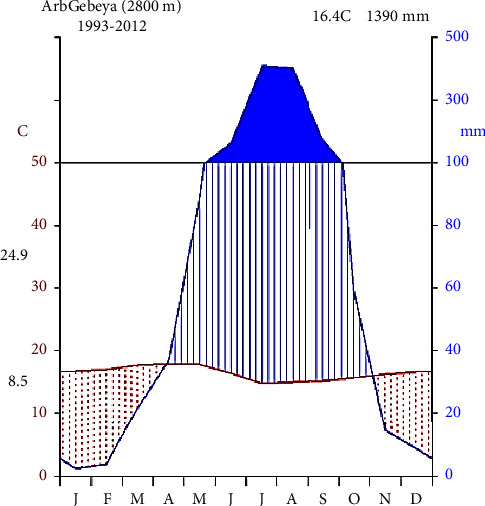
Climatic diagram of the study area at the Tach Gayint station (data source: National Meteorological Agency, 1993–2013).

Based on twenty years of weather data obtained from the National Meteorological Service Agency, the district receives 1390 mm of annual rainfall on an average, with the yearly rainfall varying between 1000 and 1600 mm across most of the district and falling primarily between May and September. The dry season runs from December to March. The average annual maximum and minimum temperatures are 24.9°C and 8.5°C, respectively (Figure 2).

### 2.3. Reconnaissance Survey and Site Selection

A reconnaissance survey was conducted from September 2 to September 30, 2020, to create a mental picture of the research area and type of vegetation, natural resources, and indigenous knowledge associated with WEPs attributes. Five kebeles (Agate, Eferata, Beteyohanes, Aneseta, and Aduka) were purposively chosen from 16 kebeles because they had good vegetation cover and the availability of WEPs. Based on the recommendations of elders, farmers, and locals, the fundamental information for site selection was obtained from the Tach Gayint district before conducting the field study.

### 2.4. Ethnobotanical Data Collection

Above the age group of 16, a total of 175 informants (119 males and 56 females) were nominated from five kebeles. In order to gather information about the perception, use, management, threats, and conservation of WEPs as well as the overall interaction between people and plants, 30 individuals from each of the five kebeles were randomly chosen by the lottery method from a list of each kebeles' inhabitants. From each of the five study kebele administrations, five key informants were purposively selected with the assistance of administrators and elders. Older people, WEP collectors, sellers, cookers, and buyers were among the key informants. Data collection methods included semistructured interviews, field observations, and focus group discussion (FGD). All informants participated in semistructured interviews to collect ethnobotanical data [22]. Interviews with local experts were arranged for the month of October, and two rounds of fieldwork in November and December were used to collect ethnobotanical data. A list of questions that were addressed in the discussion with the informants was developed in a specific order. The interviewer was asked to cover the main points on the checklist while giving the flexibility to explore any relevant issues raised by the interviewee. The researcher conducted all the interviews with the local's language in Amharic.

On the basis of the informants' level of interest, the location and the time of the talk were chosen. All relevant data, including vernacular names, habits, habitats, parts used, collection methods, and modes of consumption, as well as the strategies employed by the informants for the conservation of WEPs and the preservation of indigenous knowledge in these food plants, were recorded during guided field interviews with the informants. Field observations were made with the help of local guides and interviewees in the study area. Focus group discussions (composed of informants from each of the five kebeles) were conducted by choosing seven participants from a variety of groups, including elders, men, women, children, and other people, to gather triangulated information on WEPs as they came to an agreement. The information gathered through group discussions was useful in comparing the data collected through semistructured interviews. Before and throughout the collection of ethnobotanical data collection, brief focus group talks were held [23].

### 2.5. Voucher Specimen Collection and Identification

With the help of informants and local field assistants, a voucher specimen collection was carried out. In this period, fieldwork operations were documented, including notes on the flora and the accompanying native knowledge. In the field, photographs were also taken to document the locations, the components of the plants, and other important information. The Flora of Ethiopia and Eritrea's taxonomy keys were used for specimen identification [24].

### 2.6. Data Analysis

The ethnobotanical data were compiled and entered into an Excel spreadsheet. For quantitative information such as edible parts, growth forms, harvesting techniques, and habitats where the majority of WEPs are located, descriptive analysis was carried out using descriptive statistical techniques such as the percentage and Statistical Package for the Social Sciences (SPSS). Direct matrix ranking was used to identify the multipurpose uses of WEPs that were frequently mentioned by the key informants and preference ranking was used to determine the degree of preference for WEPs.

## 3. Results

### 3.1. Diversity of Wild Edible Plants

A total of 36 species of WEPs were identified in 25 genera and 22 families. In the district with respect to the diversity of the collected species, the highest numbers of WEPs were reported in the families of Moraceae and Anacardiaceae contributing four species (11.1%) each, followed by Lamiaceae and Polygonaceae, which represent three species (8.3%) of each (Table 1).

### 3.2. Growth Forms of Wild Edible Plants

**Figure 3:**
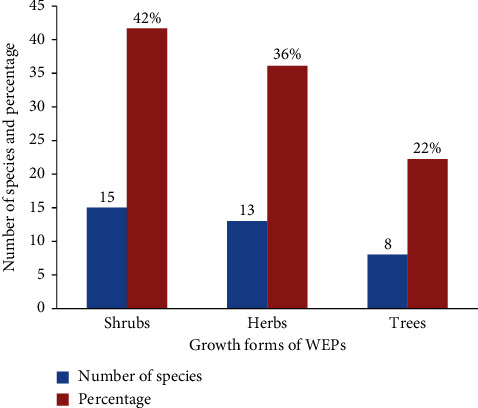
Growth forms of wild edible plants in the study area.

Among the WEPs in the study area, shrubs were the highest growth forms with 15 (42%) species, followed by herbs with 13 (36%) species (Figure 3). The result revealed that shrubs accounted for the highest proportion of growth forms.

### 3.3. Sources of Edible Wild Plants

**Figure 4:**
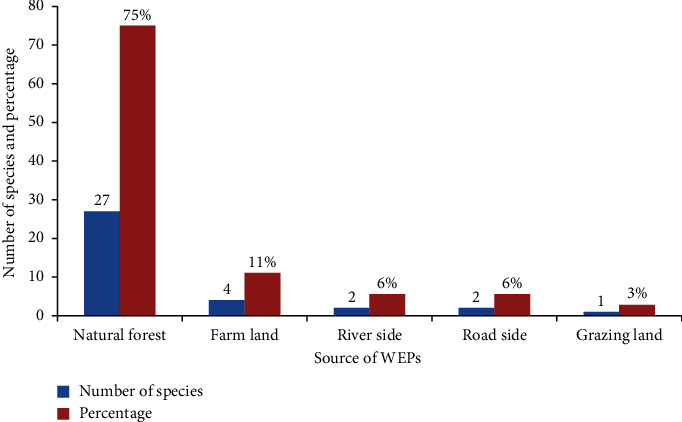
Sources of wild edible plants in the Tach Gayint district.

The edible plants were collected from various sources. The current study revealed that most (27 species, 75%) of the species of WEPs were collected from natural forests followed by farmlands (4 species, 11%) (Figure 4).

### 3.4. Plant Parts Used and Mode of Consumption

#### 3.4.1. Plant Parts Used as Food

**Figure 5:**
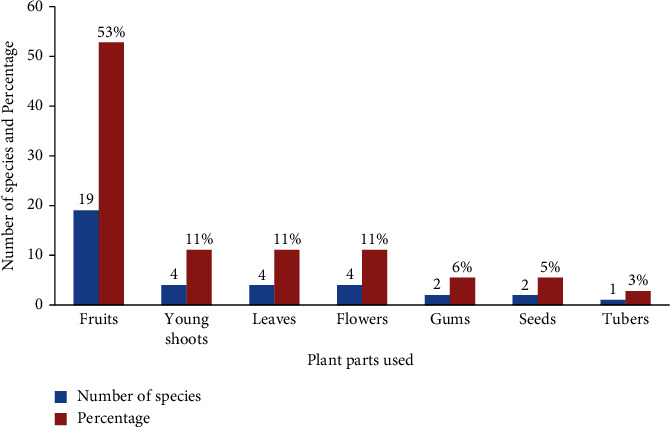
Parts of wild edible plants consumed in the study area.

In the study area, the most widely used plant part as a food includes fruits, tubers, young shoots, flowers, gums, leaves, and seeds. The most widely consumed plant parts were fruits with 19 species (53%), followed by young shoots, leaves, and flowers with 4 species (11%) each (Figure 5).

#### 3.4.2. Mode of Consumption

**Figure 6:**
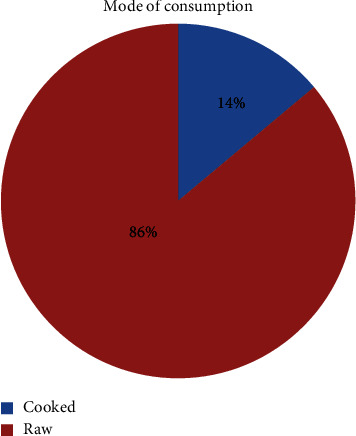
Percentage distribution of wild edible plants on mode of consumption.

Wild edible plants in the study area were consumed for supplementing staple foods and filling food gaps during drought and famine. About 31 (86%) species were reported to be eaten raw, whereas 5 (14%) species were consumed cooked or processed (Figure 6).

### 3.5. Seasonal Availability of Edible Wild Plants in the Study Area

In the study area, all of the reported wild food plants were accessible at various seasons. About 17 species (47.2%) were found in the spring season followed by 15 species (41.7%) in the fall season, while about 4 species (11.1%) of the plant species were also found in the summer season. This implies that the local community can consume at any time when famine and droughts arise; hence, it can serve as an insurance response to disasters of climate change and variability.

### 3.6. Harvesting Techniques of WEPs Reported by Informants

**Figure 7:**
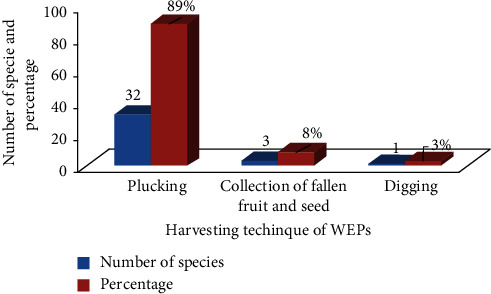
Harvesting techniques of wild edible plants in the study area.

Three primary techniques were mostly used to gather wild food plants: digging (tubers), plucking (fruits, leaves, and gums), and collecting fallen seeds and fruits from the ground. Picking from mother plants, gathering from the ground, and digging were the strategies that were most prevalent (Figure 7).

### 3.7. Variation of Indigenous Knowledge on Use of WEPs

Indigenous knowledge was reported by males than females, and the difference was significant (*P* < 0.05) when the number of WEPs reported by each group was compared. The result obtained much of the knowledge of WEPs in the study area from informants of younger ages (16–39), when compared with the elder. There was a significant difference (*P* < 0.05) in the number of WEPs reported by aged members of the community (>39 years) and young aged members (16–39 years). Similarly, there was a significant difference (*P* < 0.05) in the number of WEPs that were reported by literate and illiterate informants, key informants, and general informants in the study area (Table 2).

### 3.8. Preference Ranking

Preference ranking for the five WEPs frequently used by the local community was made by ten informants. The result obtained from the preference ranking analysis showed that *Opuntia ficus-indica* was the best preferred wild edible plant based on its taste of quality perceived by informants followed by *Ficus vasta* (Table 3).

### 3.9. Direct Matrix Ranking

Direct matrix ranking was performed to evaluate the relative importance of each of the WEP species. The direct matrix ranking of the five most popular multipurpose WEP species showed that *Cordia africana* and *Acacia abyssinica* ranked 1^st^ and 2^nd^, respectively (Table 4).

## 4. Discussion

### 4.1. Diversity of Wild Edible Plants

A total of 36 species of WEPs were identified in 25 genera and 22 families. In the district with respect to the diversity of collected species, the highest numbers of WEPs were reported in the families of Moraceae and Anacardiaceae contributing to four species (11.1%), followed by Lamiaceae and Polygonaceae, which represent three species (8.3%) of each. In the Tach Gayint district, diversity of WEPs consumed in the study area was lower than that reported in other studies within Ethiopia, and it was [25] reported that 137 WEP species were used by the Konso ethnic community in Southern Ethiopia.

The diversity of WEPs in the study area was comparable to that found in a study [26] conducted in Quara District, Northwest Ethiopia, which listed 36 WEP species. The findings were quite comparable to those of [27], who reported 37 WEP species in the semiarid East Shewa zone of Ethiopia. However, there was greater diversity in the results than in the semiarid lowlands of Southern, Ethiopia [28], where 30 WEP species were reported. These differences might be explained by variations in local biota diversity, environmental variables, local customs, and indigenous culture.

### 4.2. Growth Forms of Wild Edible Plants

In the study area, shrubs accounted for the highest growth form with 15 (42%) species, followed by herbs with 13 (36%) species (Figure 3). The result indicated that shrubs accounted for the highest proportion of growth forms. This finding also resembles with the works of [29] in the Burji Segan area of Southern Nations, Nationalities, and Peoples' Region (SNNPR), Ethiopia. The higher frequency of using shrubs and herbs might be due to the fact that shrubs and herbs are leading growth forms than trees in the study area.

### 4.3. Plant Parts Used and Mode of Consumption

The most common plant parts consumed in the study area were fruits (53%) followed by young shoots, leaves, and flowers (11%), and the remaining amount (36%) accounted for gums, seeds, and tubers (Figure 5). This shows that humans have consumed parts of various species of WEPs. The result coincides with the finding [30] where fruits were reported to be more utilized plant parts than other plant parts in the Amaro district of Southern Ethiopia. The high percentage of raw edibles may be the result of people not being encouraged to gather and use plant parts at home in preference to plant products cultivated for food, and it may be healthy to consume in raw form. Raw fruits are regarded as good sources of nutrients that do not lose their nutrients, but if they are boiled or cooked, some important nutrients may be lost.

### 4.4. Seasonal Availability of Wild Edible Plants

According to the explanations of the studied WEPs, they were available throughout the spring and fall seasons. This indicates that the majority of them are annual species that need moisture and that the season was ideally suited for their growth and reproduction due to the fact that most WEPs produced fruits in low-moisture environments, compared to staple food crops that failed during droughts and unpredictable rainfall, and the result is in line with the finding of [31] in Chilga District, Northwestern Ethiopia: implications for food security and adaptation to climate change. *A. graecizans* and *E. abyssinicum* were only available during the summer season. Consequently, collectors of WEPs need to be aware of their seasonal availability. However, in the current study, some WEPs were picked and consumed on a regular basis when ripe. These plants also become essential famine foods during times of food shortage. For example, fruits of *F. palmate*, *F. sycomorus*, *F. vasta*, *M. kummel*, *F. sur*, *O. ficus*-*indica*, *R. natalensis*, *R. glutinosa,* and *Z. spina-christi* are among those most frequently used diets during periods of food shortage [32]. In periods of severe food scarcity, people also consumed several WEPs that were often used as livestock feed in normal times. *A. graecizans*, *E. abyssinicum*, *S. pyramidalis*, *R. abyssinicus*, and *R. nervosus* are considered typical famine food plants and were consumed during food shortage in the Tach Gayint district.

### 4.5. Harvesting Techniques of Edible Wild Plants

The most typical techniques for gathering wild food plants were shaking the plant, picking the fruit with long sticks, and pulling off the mother plants. However, when people cut off the branches of wild food plants such as *A. graecizans* and *E. abyssinicum* in order to harvest the plants, the plant's growth and survival get severely affected. The practice of digging and removing tubers is another harmful consequence. This finding is consistent with a study [33] on edible wild plants used by local communities and around particular forest reserves in the Teso-Karamoja Region of Uganda.

### 4.6. Preference Ranking

The choice of WEPs varies; and some of them are consumed only during times of scarcity and not during other times. Each person has a different predilection to eat edible wild plants [34]. Every plant has different conditions or times when it is consumed. Others are only taken during times of great food scarcity and shortage, but some plants are regularly consumed even when there is a significant food supply [35]. During times of food scarcity, plants that are regularly consumed are highly valued at all levels of the society. Most of the WEPs were eaten as extra food rather than as regular meals [36]. According to the ranking of the top five WEPs by the ten key informants, *O. ficus*-*indica* stood first followed by *F. vasta*, *Z. spina*-*christi*, *F. sur*, and *U. simensis*, respectively. This shows that the abovementioned plant species have been identified as socially acceptable and significant in the study area due to their frequent consumption by a large number of users, as well as their good taste quality (Table 3).

### 4.7. Direct Matrix Ranking

The results of a direct matrix ranking exercise revealed that *C. africana*, *A. abyssinica*, and *C. spinarum* were the top three multipurpose WEPs in the study area (Table 4). As indicated in Table 4 of the findings, these plants are more adversely affected by their nonfood applications than by the reported human food values. The factors that contributed most to the extinction of the species in the study area were the overuse of multipurpose wild food plant species for things such as fuel wood, charcoal production, agricultural equipment, and fencing. The findings of this study suggest immediate preservation measures to protect rapidly declining species of multipurpose WEPs in the study area. This demonstrates how the locals have used the multipurpose plant species in a variety of ways to meet their basic needs, and these findings are comparable to those of a study conducted by [37] in the buffer area of Awash National Park, Ethiopia.

### 4.8. Indigenous Knowledge Variation on Use of WEPs

The male informants in the study area were more knowledgeable than the female informants. This might be related to the differences in occupation, including men's great attraction to WEPs during caw keeping and wood collection for house construction. This contradicts the study by [38] in the Chelia District of West-Central Ethiopia, which found that women were more knowledgeable than men. When compared to older persons, younger informants had a better understanding of WEPs in the study area. In this situation, it is possible that people who spend the most of their time herding cattle come into contact with several WEPs in this situation. They might have developed a better liking and knowledge of the plants if they had spent more time with them. The result is consistent with the findings [39] found at Dheeraa Town in Arsi, Ethiopia. This significant difference between literate, illiterate, and key informants could be attributed to the influence of experience and the maximum degree of knowledge acquisition of wild edible plants in illiterate and key informants, as well as the impact of modernization on literate communities (Table 2).

### 4.9. Threats and Conservation of Wild Edible Plants

As natural vegetation is destroyed, the habitats of these important wild food plants are becoming increasingly threatened. The WEP species, *C. benghalensis*, *C. africana*, *D. abyssinica*, *E. racemosa*, *F. communis*, *F. palmata*, *F. sycomorus*, *F. vasta*, *M. kummel*, *O. ficus-indica*, *P. peruviana*, and *P. reclinata*, were listed on the IUCN Red List of Threatened Species in this study. Due to habitat destruction and overharvesting, most WEPs face a serious threat to their survival. According to this study, anthropogenic factors such as agricultural development, gathering of firewood, overgrazing, charcoal production, and fencing have all contributed to the decline of WEP species. The loss of accessibility to WEPs has been mainly brought about by agricultural expansion. Similar findings were reported by [40] in the semiarid lowlands of southern Ethiopia. According to the informants' responses, the lack of a community institution, a conservation advisor, planting material (seedlings), infertile soil, and drought are some of the factors that impede the development of conservation practices in the study area, leading to fewer conservation efforts on WEPs in the area. The challenges listed in this study are comparable to those mentioned by [41], which include knowledge, storage, and seasonality of fruits. Due to a lack of conservation experts, local inhabitants were unaware of the potential of native wild food plants or how to add value by processing different products to improve rural communities' nutritional status, health, income, and livelihoods. It is intended that increasing public understanding of the significance of WEPs and the farming practices that produce them will encourage their preservation and long-term use.

## 5. Limitation of the Study

Due to a lack of funding for this study, the proximate composition, mineral contents, antinutritional components, and phytochemical analyses of each plant were not completed.

### 5.1. Conclusions

In the study area, a total of 36 WEP species have been identified. Most of the WEP species that were found to be edible in the study area are used by the inhabitants for a range of other things in addition to food and medicine. People in the area pick the wild plants for food, but they also use them to make furniture, building materials, and firewood. Particularly in times of food scarcity and as a supplement to cultivated plants, these wild edibles are popular with consumers. Local communities are the custodians of the native knowledge needed to utilise these WEPs. The community uses the majority of the identified WEP species in the study area for a range of uses beyond eating. The region's multifunctional WEPs are now in danger. In the study area, there are several threats to WEP species, but the government has not taken sufficient action; and there is no local conservation organisation or focal person. In addition, there was no ecological management plan in place, and the study site did not adhere to the guidelines. Due to frequent (daily) visits by residents of nearby villages for their daily needs of fuel (charcoal), agricultural expansion, and construction wood, among other things, the WEP resources are currently being depleted at a rapid rate.

### 5.2. Recommendations

The findings highlight the need for more investigation into the nutritional composition and processing methods of each species listed, as well as pharmacological properties of species used in nutraceuticals because they are also used in medicine. It is vital to investigate any potential drawbacks to using these wild foods. Improved in situ conservation of WEPs should result from the participation of the local community. Some highly valuable wild food plants are being overexploited as a result of their use; therefore, a conservation strategy should be created and implemented for the long-term management of plants.

## Figures and Tables

**Table 1:** List of wild edible plants, habit, part used, and mode of consumption in Tach Gayint district.

No.	Scientific name	Family	Local name (Amharic)	Habit	Pu	Mode of consumption and preparation	Voucher number
1	*Acacia abyssinica*	Fabaceae	Bazra Girar	T	G	Chewed by gum	MA05
2	*Acacia seyal*	Fabaceae	Nech Girar	T	G	Chewed by gum	MA07
3	*Acanthus polystachius*	Acanthaceae	Kusheslia	S	Fl	The juice of flowers' nectar is sucked by children lip	MA04
4	*Acanthus sennii*	Acanthaceae	Kusheslia	S	Fl	The juice of flowers' nectar is sucked by children lip	MA06
5	*Amaranthus graecizans*	Amaranthaceae	Aluma	H	L	Fresh leaves are boiled and eaten	MA029
6	*Becium grandiflorum*	Lamiaceae	Mutise	S	FL	The juice of the flower was squeezed with the young shoot of *Rumex nervosus* and eaten	MA026
7	*Carissa spinarum*	Apocynaceae	Agam	S	F	Fresh ripen fruits are eaten	MA028
8	*Commelina benghalensis*	Commenlinaceae	Yergn kolo	H	T	Fresh raw tuber are eaten	MA027
9	*Cordia africana*	Boraginaceae	Wanza	T	F	Raw and ripe fruits are eaten	MA03
10	*Dovyalis abyssinica*	Flacourtiaceae	Koshim	S	F	Raw and ripe fruits are eaten	MA034
11	*Erucastrum abyssinicum*	Brassicaceae	Wefe	H	L	Leaves are cooked and eaten as vegetable	MA031
12	*Euclea racemosa*	Ebenaceae	Dedeho	S	F	Raw and ripe fruits are eaten	MA030
13	*Ferula communis*	Apiaceae	Dog	H	Ys	Young shoots are rubbed/roasted with the leaf of *Rumex nervosus* and eaten	MA032
14	*Ficus palmata*	Moraceae	Beles	T	F	Raw and ripe fruits are eaten	MA033
15	*Ficus sycomorus*	Moraceae	Bamba	T	F	Raw, ripe, and dried fruits are eaten	MA024
16	*Ficus vasta*	Moraceae	Warka	T	F	Raw, ripe, and dried fruits are eaten	MA016
17	*Ficus sur*	Moraceae	Shola	T	F	Fresh raw fruits are eaten	MA01
18	*Mimusops kummel*	Sapotaceae	Eshe	T	F	Ripe and dried fruits are eaten	MA017
19	*Opuntia ficus-indica*	Cactaceae	Kulkual	S	F	Raw and ripe are pilled and eaten	MA018
20	*Phoenix reclinata*	Arecaceae	Zembaba	S	F	Raw and ripe fruits are eaten	MA019
21	*Physalis peruviana*	Solanaceae	Nech Awat	H	F	Fresh and ripe fruits are eaten	MA020
22	*Rhus natalensis*	Anacardiaceae	Takima	S	F	Raw and ripe fruits are eaten	MA021
23	*Rhus retinorrhoea*	Anacardiaceae	Talo	S	F	Raw and ripe fruits are eaten	MA022
24	*Rhus glutinosa*	Anacardiaceae	Imbis	S		Raw and ripe fruits are eaten	MA023
25	*Rosa abyssinica*	Rosaceae	Kega	S	F	Raw, ripe, and sun-dried fruits are eaten	MA025
26	*Rumex nepalensis*	Polygonaceae	Tult	H	Ys	The raw young stem is pilled and eaten	MA09
27	*Rumex nervosus*	Polygonaceae	Embacho	S	Ys	The raw young stem is pilled and eaten	MA08
28	*Rumex abyssinicus*	Polygonaceae	Mokmoko	H	Ys	The liquid is sucked from the young stem	MA010
29	*Rhus vulgaris*	Anacardiaceae	Qmmo	S	F	Raw and ripe fruits are eaten	MA011
30	*Salvia schimperi*	Lamiaceae	Gimeketle	H	FL	Flower juice is sucked by the children lip	MA013
31	*Snowdenia polystachya*	Poaceae	Muja	H	Se	The roasted and ground seed is made into injera	MA012
32	*Solanum nigrum*	Solanaceae	Tikur-awitt	H	F	Raw and ripe fruits are eaten	MA014
33	*Sporobolus pyramidalis*	Poaceae	Mure	H	Se	Seed is ground to powder and baked into injera	MA030
34	*Thymus schimperi*	Lamiaceae	Tosign	H	L	The leaves are powdered and are mixed with tea	MA015
35	*Urtica simensis*	Urticaceae	Sama	H	L	Leaves cooked as stew	MA036
36	*Ziziphus spina-christi*	Rhamnaceae	Giba	S	F	Raw fruit pulp is eaten	MA024

H, herb; S, shrub; T, tree; Pu, part used; L, leaf; F, fruit; Se, seed; FL, flower; Ys, young shoot; G, gum; T, tuber.

**Table 2:** Knowledge variation of Tach Gayint community on wild edible plants.

Parameters	Informant groups	*N*	Mean ± SD	*t* value	*P* value
Gender	Female	56	2.73 ± 2.14	5.53	0.001
	Male	119	4.72 ± 2.25		

Age	16–39	88	4.54 ± 2.35	−2.27	0.024
	>39	87	3.75 ± 2.38		

Literacy	Illiterate	138	4.34 ± 2.32	2.84	0.005
	Literate	37	3.10 ± 2.45		

Informant category	Key informant	25	6.80 ± 1.29	−6.87	0.001
	General informant	150	3.63 ± 2.23		

Significant difference (*P* < 0.05), *t* (0.05), DF = 173; *N* = number of respondents.

**Table 3:** Result of preference ranking of five selected WEPs based on their taste and frequent consumption by the informants in the study area.

WEP species	Preference ranking of selected wild edible plants											
	*R*_1_	*R*_2_	*R*_3_	*R*_4_	*R*_5_	*R*_6_	*R*_7_	*R*_8_	*R*_9_	*R*_10_	Total	Rank
*Ficus sur*	1	2	3	2	1	2	1	3	3	1	19	4^th^
*Ficus vasta*	2	1	3	1	2	3	1	4	2	3	22	2^nd^
*Opuntia ficus-indica*	3	1	2	3	4	5	2	5	2	1	28	1^st^
*Urtica simensis*	2	1	2	1	2	1	1	2	3	1	16	5^th^
*Ziziphus spina-christi*	2	3	2	1	1	2	3	1	2	4	21	3^rd^

1 = least; 2 = less; 3 = good; 4 = very good; 5 = excellent.

**Table 4:** Direct matrix ranking of six plant species by ten informants based on five use criteria.

Use categories	Direct matric ranking of selected wild edible plants						
	*Acacia abyssinica*	*Carissa spinarum*	*Cordia africana*	*Ficus vasta*	*Rosa abyssinica*	Total	Rank
Charcoal	4	2	3	3	0	12	5^th^
Medicine	2	3	2	1	3	11	6^th^
Fire wood	4	3	3	2	4	16	1^st^
Agricultural tool	3	2	5	2	1	13	4^th^
Fence	4	3	3	1	3	14	3^rd^
Food	1	3	4	4	3	15	2^nd^
Total	18	16	20	13	14		
Rank	2^rd^	3^nd^	1^st^	5^th^	4^th^		

5 = best; 4 = very good; 3 = good; 2 = less used; 1 = least used; 0 = no value.

## Data Availability

The data used to support the findings of this study are included within the article.
